# Supplementary material for: Polyketide synthase gene clusters in the ericoid mycorrhizal fungus Oidiodendron maius: genome-wide prediction, expression profiling, and functional characterization of knockout mutants
Source: Front Plant Sci. 2026 Sep 16;17:1924430. doi: 10.3389/fpls.2026.1924430 (PMC13623903; doi:10.3389/fpls.2026.1924430)
Supplement: Supplementary file 1 [file SupplementaryFile1.zip › Supplementary Materials/Supplementary Folder 1.zip!/Oidma1_AssemblyScaffolds_Repeatmasked/knownclusterblast/cluster58/jgi.p_Oidma1_62281_mibig_hits.html]

| MIBiG Protein | Description | MIBiG Cluster | MiBiG Product | % ID | % Coverage | BLAST Score | E-value |
| --- | --- | --- | --- | --- | --- | --- | --- |
| AAK83183.1 | putative\_NDP-glucose\_4,6-dehydratase | BGC0000026 | Avilamycin\_A\_biosynthetic\_gene\_cluster | 48 | 97.7917981073 | 274 | 1.7e-73 |
| ABC02802.1 | putative\_UDP-glucose\_4-epimerase | BGC0000809 | AT2433\_biosynthetic\_gene\_cluster | 34 | 99.6845425868 | 176 | 3.6e-44 |
| ABY66025.1 | glucuronic\_acid\_decarboxylase | BGC0001008 | Maduropeptin\_biosynthetic\_gene\_cluster | 34 | 100.94637224 | 165 | 8.3e-41 |
| BAP34705.1 | NAD-dependent\_epimerase/dehydratase | BGC0000078 | Incednine\_biosynthetic\_gene\_cluster | 33 | 102.208201893 | 158 | 1.3e-38 |
| AAM70333.1 | CalS9 | BGC0000033 | Calicheamicin\_biosynthetic\_gene\_cluster | 32 | 101.577287066 | 148 | 1.8e-35 |
| AAD13546.1 | NDP-hexose\_4,6-dehydratase\_homolog | BGC0000239 | Landomycin\_biosynthetic\_gene\_cluster | 29 | 101.892744479 | 124 | 2.8e-28 |
| BAG50456.1 | dTDP-D-glucose\_4,6-dehydratase | BGC0000780 | O&K-antigen\_biosynthetic\_gene\_cluster | 27 | 106.309148265 | 123 | 4.7e-28 |
| ABB05110.1 | LipDig4 | BGC0001003 | Lipomycin\_biosynthetic\_gene\_cluster | 28 | 101.577287066 | 123 | 6.2e-28 |
| AAC63612.1 | RmlB | BGC0000773 | Lipopolysaccharide\_biosynthetic\_gene\_cluster | 28 | 106.940063091 | 122 | 8.1e-28 |
| AAG02361.1 | sugar\_epimerase\_BlmG | BGC0000963 | Bleomycin\_biosynthetic\_gene\_cluster | 30 | 101.892744479 | 121 | 1.4e-27 |
| AAC68681.1 | TDP-glucose-4,6-dehydratase | BGC0000898 | Desosamine\_biosynthetic\_gene\_cluster | 27 | 102.523659306 | 121 | 1.4e-27 |
| BAO66514.1 | 4-ketoreductase | BGC0000042 | Cremimycin\_biosynthetic\_gene\_cluster | 29 | 102.839116719 | 120 | 4e-27 |
| AHH99944.1 | dTDP-glucose\_4,6-dehydratase | BGC0000002 | Aculeximycin\_biosynthetic\_gene\_cluster | 28 | 102.523659306 | 120 | 4e-27 |
| AAM88357.1 | NbmH | BGC0000899 | Desosamine\_biosynthetic\_gene\_cluster | 26 | 102.523659306 | 119 | 5.2e-27 |
| AHZ61891.1 | Lom57 | BGC0000240 | Lomaiviticin\_biosynthetic\_gene\_cluster | 27 | 102.523659306 | 119 | 8.9e-27 |
| BAC57041.1 | dTDP-glucose-4,6-dehydratase | BGC0000102 | Mycinamicin\_biosynthetic\_gene\_cluster | 28 | 102.523659306 | 118 | 1.2e-26 |
| AAX98199.1 | sugar\_dehydratase/epimerase | BGC0000052 | ECO-02301\_biosynthetic\_gene\_cluster | 27 | 102.523659306 | 118 | 1.2e-26 |
| CAI94677.1 | putative\_TDP-glucose\_dehydratase | BGC0000141 | Rubradirin\_biosynthetic\_gene\_cluster | 28 | 102.523659306 | 118 | 1.5e-26 |
| CAB01951.1 | ExpA9 | BGC0000801 | Galactoglucan\_biosynthetic\_gene\_cluster | 28 | 105.362776025 | 118 | 2e-26 |
| AAC44074.1 | dTDP-D-glucose-4,6-dehydratase | BGC0000797 | Sphingan\_polysaccharide\_biosynthetic\_gene\_cluster | 26 | 104.731861199 | 117 | 2.6e-26 |
| AGC09479.1 | LobD5 | BGC0001183 | Lobophorin\_biosynthetic\_gene\_cluster | 28 | 102.208201893 | 116 | 4.4e-26 |
| AGI99502.1 | sugar\_4,6-dehydratase | BGC0001004 | Lobophorin\_biosynthetic\_gene\_cluster | 28 | 102.208201893 | 116 | 4.4e-26 |
| ABW74897.1 | dTDP-D-glucose-4,6-dehydratase | BGC0000759 | Diutan\_polysaccharide\_biosynthetic\_gene\_cluster | 27 | 104.731861199 | 116 | 4.4e-26 |
| ACB37735.1 | dTDP-glucose\_4,6-dehydratase | BGC0000162 | Tetrocarcin\_A\_biosynthetic\_gene\_cluster | 27 | 103.154574132 | 115 | 9.9e-26 |
| CAF31365.1 | putative\_dNDP-glucose\_4,\_6-dehydratase | BGC0000268 | Sch47554\_/\_Sch47555\_biosynthetic\_gene\_cluster | 28 | 102.523659306 | 113 | 6.4e-25 |
| ACB46492.1 | sugar\_4,6-dehydratase | BGC0000082 | Kijanimicin\_biosynthetic\_gene\_cluster | 27 | 103.154574132 | 111 | 1.9e-24 |
| BAA84593.1 | dTDP-glucose\_4,6-dehydratase | BGC0000025 | Avermectin\_biosynthetic\_gene\_cluster | 28 | 101.892744479 | 111 | 1.9e-24 |
| ABX00609.1 | LmbM | BGC0000907 | Lincomycin\_biosynthetic\_gene\_cluster | 31 | 83.2807570978 | 107 | 3.5e-23 |
| ADB92562.1 | CcbM | BGC0001225 | Celesticetin\_biosynthetic\_gene\_cluster | 29 | 82.9652996845 | 103 | 3.9e-22 |
| AAG26471.1 | putative\_UDP-glucuronic\_acid\_epimerase | BGC0001411 | Polysaccharide\_B\_biosynthetic\_gene\_cluster | 26 | 112.933753943 | 103 | 5.1e-22 |
| AAK83169.1 | putative\_UDP-glucose\_4-epimerase | BGC0000026 | Avilamycin\_A\_biosynthetic\_gene\_cluster | 28 | 99.0536277603 | 101 | 1.5e-21 |
| AAA64648.1 | type\_1\_capsule\_synthesis\_gene | BGC0000758 | Capsular\_polysaccharide\_biosynthetic\_gene\_cluster | 26 | 109.148264984 | 101 | 1.9e-21 |
| ADG29285.1 | putative\_UDP-glucose\_4-epimerase | BGC0000796 | S-layer\_glycan\_biosynthetic\_gene\_cluster | 26 | 102.523659306 | 97 | 3.6e-20 |
| AAD44205.1 | GepiA | BGC0000362 | Glycopeptidolipid\_biosynthetic\_gene\_cluster | 24 | 111.041009464 | 97 | 3.6e-20 |
| ABK27324.1 | GalE | BGC0000790 | O-antigen\_biosynthetic\_gene\_cluster | 32 | 82.9652996845 | 95 | 1.1e-19 |
| CAJ77705.1 | RmlB\_protein | BGC0000364 | Glycopeptidolipid\_biosynthetic\_gene\_cluster | 25 | 111.987381703 | 94 | 2.4e-19 |
| AAK83179.1 | putative\_NDP-glucose\_4-epimerase | BGC0000026 | Avilamycin\_A\_biosynthetic\_gene\_cluster | 28 | 82.0189274448 | 88 | 1.3e-17 |
| CAF31840.1 | putative\_NDP-heptose/hexose\_dehydrogenase | BGC0000699 | Hygromycin\_B\_biosynthetic\_gene\_cluster | 27 | 100.94637224 | 85 | 1.4e-16 |
| AAR90883.1 | UDP-glucose\_C4-epimerase | BGC0000787 | O-antigen\_biosynthetic\_gene\_cluster | 26 | 105.678233438 | 82 | 1.2e-15 |
| AAL25635.1 | UDP-galactose\_4-epimerase | BGC0000786 | O-antigen\_biosynthetic\_gene\_cluster | 28 | 80.1261829653 | 77 | 2.3e-14 |
| CAI47661.1 | putative\_epimerase/dehydratase | BGC0000692 | Apramycin\_biosynthetic\_gene\_cluster | 26 | 99.3690851735 | 73 | 5.6e-13 |
| ACA14473.1 | HldD | BGC0000778 | Lipopolysaccharide\_biosynthetic\_gene\_cluster | 24 | 86.7507886435 | 64 | 2.6e-10 |
| AHX24686.1 | NAD-dependent\_epimerase/dehydratase | BGC0000200 | Arixanthomycin\_biosynthetic\_gene\_cluster | 30 | 41.3249211356 | 55 | 1.6e-07 |
| AAM27548.1 |  | BGC0000784 | O-antigen\_biosynthetic\_gene\_cluster | 26 | 90.2208201893 | 55 | 1.6e-07 |
| ACO05923.1 | GalE | BGC0000763 | Stewartan\_biosynthetic\_gene\_cluster | 46 | 20.5047318612 | 51 | 2.3e-06 |
